# Supplementary material for: Seasonal Variations in Stroke and a Comparison of the Predictors of Unfavorable Outcomes among Patients with Acute Ischemic Stroke and Cardioembolic Stroke
Source: Biomedicines. 2024 Jan 19;12(1):223. doi: 10.3390/biomedicines12010223 (PMC10813505; doi:10.3390/biomedicines12010223)
Supplement: Supplementary file 1 [file biomedicines-12-00223-s001.zip › biomedicines-2755575-supplementary.pdf]

Table S1. Univariate analysis of factors influencing unfavorable outcomes (mRS >2) in all 4,040 patients with AIS and 729 patients with CE stroke

Characteristics	All 4040 patients with AIS			729 patients with CE stroke		
	No (n =1778)	Yes (n = 2262)	*p*-Value	No (n =229)	Yes (n = 500)	*p*-Value
Age (years)	66.4 (57.8–75.4)	76.9 (66.3–84.5)	<0.001	72.7 (65.8–81.3)	80.3 (71.8–87.3)	<0.001
SBP (mmHg)	161 (143–184)	161 (141–185)	0.809	154 (137–176)	158 (136–181)	0.335
Heart rate (beats/minute)	79 (68–90)	81 (70–93)	<0.001	85 (72–98)	85 (71–99)	0.795
Initial NIHSS score	3 (2–4)	9 (5–16)	<0.001	3 (2–5)	15 (7–23)	<0.001
Hemoglobin (g/dL)	14.1 (12.8–15.3)	13.4 (11.9–14.7)	<0.001	14.0 (12.9–15.4)	13.3 (11.8–14.7)	<0.001
Platelet ($\times 10^9$/L)	211 (175–256)	205 (165–253)	<0.001	194 (156–231)	188 (153–231)	0.855
NLR	2.6 (1.8–4.0)	3.3 (2.1–5.6)	<0.001	2.6 (1.9–4.2)	3.4 (2.1–6.4)	<0.001
Glucose (mg/dL)	134 (109–183)	139 (115–193)	<0.001	125 (108–160)	135 (114–174)	0.024
Creatinine (mg/dL)	1.01 (0.87–1.30)	1.08 (0.88–1.40)	0.081	1.01 (0.90–1.30)	1.10 (0.90–1.40)	0.296
LDL-cholesterol (mg/dL)	106 (85–129)	102 (80–127)	<0.001	93 (76–111)	92 (72–115)	0.554
Triglyceride (mg/dL)	113 (80–161)	96 (70–139)	<0.001	87 (60–122)	79 (59–109)	0.032
Uric acid (mg/dL)	5.3 (4.2–6.2)	5.0 (4.0–6.2)	0.024	5.5 (4.3–6.6)	5.3 (4.2–6.6)	0.024
Female gender	603 (34)	1138 (50)	<0.001	92 (40)	274 (55)	<0.001
Hypertension	1233 (69)	1630 (72)	0.065	157 (69)	357 (71)	0.435
Diabetes mellitus	583 (33)	884 (39)	<0.001	58 (25)	163 (33)	0.045
Heart disease	407 (23)	761 (34)	<0.001	174 (76)	381 (76)	0.949
Dyslipidemia	446 (25)	432 (19)	<0.001	60 (13)	52 (10)	0.313
Prior stroke	333 (19)	629 (28)	<0.001	52 (23)	138 (28)	0.174
Current smoker	502 (28)	351 (16)	<0.001	42 (18)	52 (10)	0.004
Alcohol consumption	151 (8)	106 (5)	<0.001	19 (8)	16 (3)	0.005
Cancer history	90 (5)	185 (8)	<0.001	17 (7)	44 (9)	0.568
In-hospital complications	20 (1)	489 (22)	<0.001	5 (2)	164 (33)	<0.001
Neurological deterioration	44 (2)	381 (17)	<0.001	3 (1)	100 (20)	<0.001

AIS, acute ischemic stroke; CE, cardioembolic; NIHSS, NIHSS, National Institutes of Health Stroke Scale; NLR, neutrophil-to-lymphocyte ratio; SBP, systolic blood pressure

Table S2. Univariate analyses of death predictors in all 4,040 patients with AIS and 729 patients with CE stroke

Characteristics	All 4040 patient with AIS			729 patients with CE stroke		
	No (*n* = 3849)	Yes (*n* = 191)	*p*-Value	No (*n* = 656)	Yes (*n* = 73)	*p*-Value
Age (years)	71.5 (61.9–81.1)	80.1 (68.2–87.6)	<0.001	77.8 (68.8–85.1)	81.4 (72.8–89.7)	0.003
SBP (mmHg)	161 (142–184)	169 (140–194)	0.140	156 (136–177)	170 (137–194)	0.062
Heart rate (beats/minute)	80 (69–91)	87 (74–100)	<0.001	85 (71–98)	89 (76–105)	0.047
Initial NIHSS score	4 (2–9)	22 (15–28)	<0.001	8 (3–17)	23 (17–27)	<0.001
Hemoglobin (g/dL)	13.8 (12.4–15.0)	13.2 (11.3–14.9)	<0.001	13.6 (12.2–15.0)	12.9 (11.5–14.3)	0.078
Platelet ($\times 10^9$/L)	208 (170–254)	207 (157–253)	0.223	189 (154–229)	202 (160–256)	0.180
NLR	2.9 (2.0–4.6)	4.6 (2.5–9.7)	<0.001	3.0 (2.0–5.4)	4.3 (1.9–9.7)	0.009
Glucose (mg/dL)	136 (112–187)	155 (125–216)	<0.001	132 (112–165)	147 (122–204)	0.006
Creatinine (mg/dL)	1.01 (0.86–1.30)	1.20 (0.94–1.62)	<0.001	1.10 (0.90–1.40)	1.20 (0.90–1.67)	0.073
LDL-cholesterol (mg/dL)	104 (83–128)	94 (68–122)	0.002	93 (74–113)	92 (61–119)	0.754
Triglyceride (mg/dL)	113 (80–161)	96 (70–139)	<0.001	82 (60–115)	78 (62–95)	0.437
Uric acid (mg/dL)	5.1 (4.1–6.2)	5.2 (4.1–7.2)	0.089	5.4 (4.3–6.5)	5.4 (4.6–7.8)	0.067
Female gender	1647 (43)	94 (49)	0.085	326 (50)	40 (55)	0.459
Hypertension	2733 (71)	129 (68)	0.328	465 (71)	49 (67)	0.501
Diabetes mellitus	1402 (36)	65 (34)	0.538	195 (30)	26 (36)	0.347
Heart disease	1066 (28)	102 (53)	<0.001	495 (75)	60 (82)	0.247
Dyslipidemia	855 (22)	23 (12)	<0.001	78 (12)	4 (5)	0.118
Prior stroke	927 (24)	35 (18)	0.068	174 (27)	16 (22)	0.482
Smoking	822 (21)	30 (16)	0.069	89 (14)	5 (7)	0.139
Alcohol consumption	250 (6)	7 (4)	0.129	35 (5)	0 (0)	0.040
Cancer history	249 (6)	26 (14)	<0.001	46 (7)	15 (21)	<0.001
In-hospital complications	416 (11)	93 (49)	<0.001	131 (20)	38 (52)	<0.001
Neurological deterioration	341 (9)	84 (44)	<0.001	70 (11)	33 (45)	<0.001

AIS, acute ischemic stroke; CE, cardioembolic; NIHSS, NIHSS, National Institutes of Health Stroke Scale; NLR, neutrophil-to-lymphocyte ratio; SBP, systolic blood pressure
